# Demographic Profiles and Methodologies Used in the Generation and Validation of Resting Metabolic Rate Prediction Equations: Protocol for a Systematic Review

**DOI:** 10.2196/82482

**Published:** 2026-04-30

**Authors:** James W Navalta, Jafrā D Thomas, Whitley J Stone

**Affiliations:** 1 Department of Kinesiology and Nutrition Sciences University of Nevada, Las Vegas Las Vegas, NV United States; 2 Department of Kinesiology and Public Health California Polytechnic State University, San Luis Obispo San Luis Obispo, CA United States; 3 School of Kinesiology, Recreation, and Sport Western Kentucky University Bowling Green, KY United States

**Keywords:** resting metabolic rate, prediction equations, demographic characteristics, gender nonbinary, health equity, physical activity

## Abstract

**Background:**

Resting metabolic rate (RMR) prediction equations used today often rely on the consideration of binary sex. Significant intrasex variability and a lack of data on diverse populations raise concerns about these equations’ validity and generalizability. Existing systematic reviews have focused on specific populations like individuals with obesity or athletes, but none have systematically examined the demographic characteristics of participants used to derive these equations. Our central hypothesis is that the accuracy of RMR prediction is influenced by the demographic alignment between the equation’s derivation population and the individual. We present a systematic review protocol to critically evaluate the literature and participant demographic profiles that underpin current RMR prediction equations.

**Objective:**

Our objectives are to (1) determine the characteristics of participant populations, including reporting on gender and sex diversity, used in RMR equation research; (2) critically appraise the methodologies, findings, and reporting practices of studies that developed RMR equations for binary populations; and (3) use the Sex and Gender Equity in Research guidelines to assess sex and gender terminology and variable inclusion in the generative RMR prediction literature.

**Methods:**

Following a PROSPERO-registered protocol (CRD420251084400), we will conduct a comprehensive search across multiple databases, including Academic Search Premier, PubMed, and Web of Science. The final search string will be: ((resting metab* rate) OR (RMR) OR (basal metab* rate) OR (BMR) OR (metabol*) OR (resting energy expenditure) OR (metab* rate)) AND ((predict* equation) OR (predict* model) OR (predict* algorithm) OR (formula) OR (estimation equation)) AND ((demograph*) OR (characterist*) OR (age) OR (race) OR (ethnicity) OR (sex) OR (gender)). We will include peer-reviewed, English-language articles reporting studies that generated RMR prediction equations and reported human participant demographic characteristics. Exclusion criteria include studies not generating prediction equations, without demographic data, or involving animals. Data extraction will include reported participant demographics (eg, sex, gender, race or ethnicity, age, and body composition), RMR test protocols, and reported reliability or validity metrics. Risk of bias will be assessed using PROBAST (Prediction Model Risk of Bias Assessment Tool).

**Results:**

This study was funded in June 2025 by the University of Nevada, Las Vegas Sports Innovation Initiative Catalyst Grant Funding Program and in July 2025 by the National Association for Kinesiology in Higher Education Hellison Interdisciplinary Research Grant. The databases were searched using the final search string between August 1, 2025, and August 8, 2025. Training of team members began on September 3, 2025, and concluded on October 20, 2025.

**Conclusions:**

Findings will be disseminated through a narrative synthesis submitted for publication, adhering to the PRISMA (Preferred Reporting Items for Systematic Review and Meta-Analyses) reporting guidelines. This review will identify gaps in the inclusivity and generalizability of current RMR prediction equations, informing future research and clinical applications.

**Trial Registration:**

PROSPERO CRD420251084400; https://www.crd.york.ac.uk/PROSPERO/view/CRD420251084400

**International Registered Report Identifier (IRRID):**

PRR1-10.2196/82482

## Introduction

### Background

Many resting metabolic rate (RMR) prediction equations require the input of binary sex to return estimates [1-4]. Basing the estimate on sex may be inherently flawed, given the known high intrasex variability among cisgender male participants compared with cisgender female participants [5,6]. At issue is the ability of binary equations to return valid and reliable measures for cisgender female or male participants, let alone be used to make generalizations about resting metabolism among intersex and transgender people. The concepts of sex and gender have been historically conflated and often used interchangeably [7]. Sex typically denotes biological attributes in humans and animals associated with physical and physiological features, such as chromosomes, gene expression, hormone function, and reproductive or sexual anatomy [8]. By contrast, gender refers to the socially constructed roles, behaviors, and identities that are often represented by feminine, masculine, and gender-diverse people [8,9]. It is unclear to what extent formative evaluations have been undertaken to determine the generation of current prediction equations for a variety of population subgroups.

Several systematic reviews have been conducted on resting metabolism and prediction equations [10-14]. Many have focused on the ability of RMR prediction equations to return accurate values in specific populations, such as people with obesity [11,12,14] and those who are critically ill [13]. A review specific to prediction in athletes determined that while no single equation is superior, measurement accuracy can be increased by choosing an equation that aligns most similarly with the characteristics of the athlete [10]. Our central hypothesis is that this finding would extend to people who identify as gender-diverse. To our knowledge, the literature has not been systematically reviewed to determine the demographic characteristics of individuals who participated in the derivation of RMR prediction equations.

### Study Aims

We aim to critically evaluate the available literature and participant demographic profiles around RMR that have led to the energy estimation equations currently used. There is a need to (1) determine characteristics of participant populations used in research to generate resting metabolism equations, including any reporting about participants’ gender and sex diversity; (2) critically appraise the methodologies, findings, and reporting practices of original studies used to develop resting metabolism equations for binary gender and sex populations; and (3) use the Sex and Gender Equity in Research (SAGER) guidelines [15] to determine whether terminology, reporting, and interpretations regarding sex and gender are used appropriately in the literature used to generate RMR prediction equations.

## Methods

### Overview

Scoping searches suggested there are no systematic reviews describing demographic characteristics of participants in studies used to generate RMR prediction equations. The authors consulted peer-reviewed guidance about conducting systematic reviews [16] and have previously participated in the process [17-24]. Following deliberation, the study investigators chose to adapt the population, exposure, comparator, outcome, and study design (PECOS) framework to structure this review protocol, which is available in PROSPERO [25]. The PECOS framework (illustrated in Table 1) was used to formalize the review’s search parameters and delineate which details to extract from included articles.

**Table 1:** Population, exposure, comparator, outcome, and study design (PECOS) study table.

PECOS element	Description
Population	Adults or children
Exposure	Predictive attributes (eg, anthropometric and demographic variables, such as age, sex, height, weight, body composition [including lean body mass, and fat mass], and/or physiological variables such as heart rate) used for deriving RMR^a^ prediction equations
Comparator	RMR measured by indirect calorimetry
Outcome	Accuracy of study-generated RMR prediction equation Precision of study-generated RMR prediction equation
Study design	Cross-sectional studies

^a^RMR: resting metabolic rate.

Studies will be described by their sample’s demographic characteristics, the predictor variables comprising the finalized RMR prediction equations, any established equation the new prediction equation was compared with, the method by which indirect calorimetry was measured, and whether precise estimates of accuracy and precision are reported. Exposure variables in RMR prediction equation research are demographic (eg, age and sex) and anthropometric variables (eg, height, weight, and lean body mass). For purposes of this review, accuracy and precision are used as proxy measures of prediction equation validity and reliability. In the RMR literature, accuracy is defined as the percentage point difference between predicted and measured RMR [10]. Precision is defined as the number of observations within the study sample that are within a specified percentage point range of participants’ measured RMR (eg, ≤10% difference) [10]. That said, the research team will document other measures of validity and reliability reported in articles describing generative RMR equations. Regarding study design, the typical design is expected to be cross-sectional. However, other study designs used to generate an RMR equation will be documented. Regarding sampling procedures, no restrictions will be imposed. Studies may use randomized, purposive, or convenience sampling, and these procedures will be documented.

The protocol for the systematic review will adhere to the following steps: (1) determine the review question and PECOS criteria, (2) create inclusion and exclusion criteria, (3) create and follow a search strategy, (4) document sources that are included and excluded according to the predetermined eligibility criteria, (5) assess final sources for risk of bias, (6) extract pertinent data from final full-text articles and synthesize the information, and (7) disseminate findings of the systematic review. During this process, documentation of the inclusion and exclusion of records will be maintained. Clear documentation allows the systematic review to be transparent and reproducible, an attribute that distinguishes it from traditional literature reviews [26].

### Search Strategy

The search terms for RMR will include: “resting metab* rate,” “RMR,” “basal metab* rate,” “BMR,” “metabol*,” “resting energy expenditure,” and “metab* rate.”

Other terms associated with RMR include “standard metab* rate,” “minimum metabolism,” and “sleeping metab* rate.” However, upon pilot testing, these terms did not add to the search results and were deleted.

The search terms for prediction equations will include: “predict* equation,” “predict* model,” “predict* algorithm,” “formula,” “estimation equation,” “estimation formula,” “prognostic model,” “pred* analytics,” “deriv* equation.”

The search terms for demographic measures will include: “demograph*,” “characterist*,” “age,” “race,” “ethnicity,” “sex,” and “gender.”

Other terms associated with sex and gender include “nonbinary,” “non-bina*,” “transgend*,” “gender queer,” “sex assign*,” and “gender nonconform*.” However, upon pilot testing, these terms did not add to the search results and were deleted.

The final search string will be: ((resting metab* rate) OR (RMR) OR (basal metab* rate) OR (BMR) OR (metabol*) OR (resting energy expenditure) OR (metab* rate)) AND ((predict* equation) OR (predict* model) OR (predict* algorithm) OR (formula) OR (estimation equation) OR (estimation formula) OR (prognostic model) OR (pred* analytics) OR (deriv* equation)) AND ((demograph*) OR (characterist*) OR (age) OR (race) OR (ethnicity) OR (sex) OR (gender))

### Electronic Bibliographic Databases

The search will occur through the Academic Search Premier, APA PsycInfo, PubMed, Web of Science, and SPORTDiscus databases.

### Other Search Methods

The reference list of final articles for inclusion will be checked for any source that appears relevant but was missed in the initial database search; sources identified from checking the references will be screened for inclusion in accordance with the inclusion and exclusion procedures.

### Inclusion and Exclusion Criteria

The review questions and PECOS criteria guide the eligibility, inclusion, and exclusion criteria for this systematic review. These characteristics provide clarity to the review team, enabling the exclusion of irrelevant sources during the screening process. Efficient screening is important because teams may review hundreds to thousands of sources. In consultation with the review questions and PECOS criteria, the following eligibility criteria were created (Textbox 1).

**Eligibility criteria.**

**Participants**

- Apparently healthy adults and children who participated in published scientific literature that generated resting metabolic rate (RMR) equations.

**Inclusion criteria**

- The source is a published article in a peer-reviewed journal.
- The source is available in English.
- The source reports findings of an interventional or observational study that results in the generation of RMR prediction equations.
- The source reports demographic characteristics of human participants in terms of sex or gender.

**Exclusion criteria**

- The source is not a published, peer-reviewed journal article.
- The source is written in any language other than English.
- The source assesses RMR, but it does not result in a new prediction equation.
- The source does not report participant demographic characteristics of sex or gender.
- The source includes nonhuman participants (ie, animal studies).
- The source reports RMR prediction equations generated for populations with acute or chronic diseases.

### Study Screening and Selection

Sources located through the search strategy described earlier will be uploaded into the Covidence systematic review software (Veritas Health Innovation) and screened for potential inclusion in stages, per the study eligibility criteria: (1) screen based on title and abstract and (2) screen a relevant subset based on full text. The date range will not be limited due to RMR prediction studies being reported in the literature as early as 1918 [4]. The software identifies and removes duplicate sources between databases. It is possible that duplicates may be removed manually (ie, if the software has not recognized a duplicate published as a preprint).

Team members (n=10) will be trained on the systematic review inclusion and exclusion criteria using a small subset of articles (n=21). In addition to the study’s primary investigators (n=3), once a research assistant team member (n=7) has demonstrated proficient interrater agreement (Krippendorff alpha) [27] and validity screening (comparison to primary investigator consensus codes), that team member will be invited to be the primary screener of articles for inclusion in the review study. Research assistants will work independently to screen articles for inclusion in the Covidence system. Sources will be included based upon the consensus of 2 raters. One of the study’s primary investigators (n=3) will independently adjudicate disagreements. Based on past experience of our team and other investigators [28], corresponding authors rarely respond to queries. Thus, we will not attempt to contact corresponding authors for information about their study write-up or to gain access to its full text. The online interlibrary loan system of the research teams’ institutions will be used to access sources approved for full-text screening.

### Data Management

**Figure 1:**
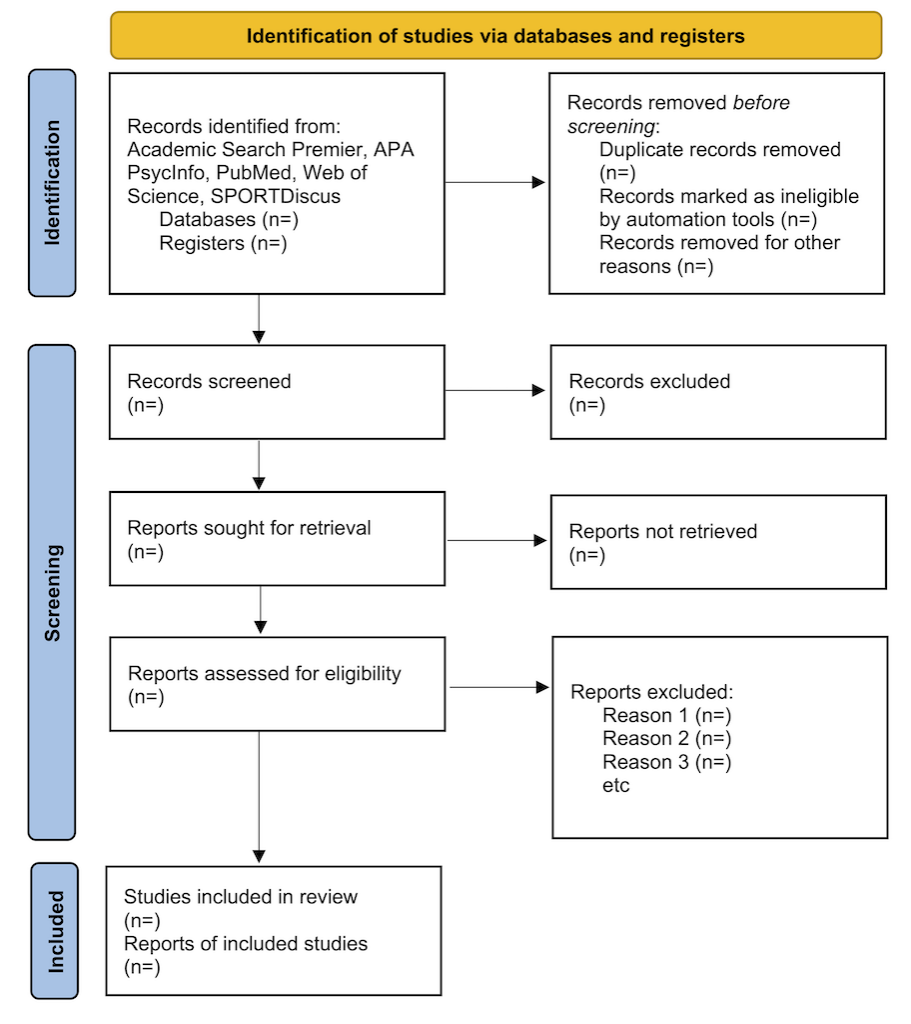
Preliminary PRISMA (Preferred Reporting Items for Systematic Reviews and Meta-Analyses) diagram that will be used for the systematic review protocol.

Data will be managed through the Covidence systematic review software noted earlier. This cloud-based software allows team members from various institutions to collaborate on the systematic review. Covidence merges the output files imported for screening from each database and removes initial duplicates. Team members will also manually remove duplicates not identified by the software. Covidence will track not only the imported data and removal of duplicates but also studies screened at the title and abstract stage, full-text stage, and data extraction stage, compiling the aforementioned data into a PRISMA (Preferred Reporting Items for Systematic Review and Meta-Analyses) flowchart (example shown in Figure 1).

### Data Extraction

Research team members will independently extract data into specifically designed extraction templates within the Covidence software. Prior to data extraction, the template will be pilot tested by the study’s primary investigators using at least 3 studies. Additionally, research assistant team members will receive training, and their extracted data will be checked against that of the study’s primary investigators. If further explanation and instruction are necessary, they will be applied at this point. Once the study’s primary investigators and research assistant team members are confident with the data extraction pilot, the data extraction phase will commence. Extraction will occur in duplicate by 2 independent team members and will be independently adjudicated by one of the study’s primary investigators.

The following data, if reported, will be extracted from the included articles: demographic profile of participants used in the generation of RMR prediction equations—sex and gender, race or ethnicity, age, height, mass, body composition, activity level, fitness level, and health condition status. For the purposes of this systematic review, we will define sex as biological attributes associated with physical and physiological features, including chromosomes, gene expression, hormone function, and reproductive or sexual anatomy. We will define gender as socially constructed roles, behaviors, and identities represented by feminine, masculine, and gender-diverse people. We acknowledge that race [29] (typically used to categorize people based on physical characteristics like skin color and hair texture) and ethnicity [30] (categorization of people who share cultural practices, including customs, language, norms, and values) are socially constructed concepts. For the purposes of this systematic review, we will extract race or ethnicity as the authors used these terms to categorize participants, if present.

Additional data to be extracted will include RMR testing protocols used to generate prediction equations—equipment, sampling duration, data processing, environmental conditions, diet prior to RMR testing, composition of meals if available, time of day testing occurred, sleep quality or duration prior to testing. Finally, any evidence of reliability (ie, intraclass correlation coefficient and coefficient of variation) or validity and agreement metrics (ie, mean absolute percent error, Lin’s Concordance Correlation Coefficient, Pearson product-moment correlation, bias, and limits of agreement) of the prediction equation, and whether the RMR method was based on cited literature, will be extracted. Once all data are extracted, a narrative synthesis of the extracted data will be written to report the main findings and implications. The synthesis will include an assessment of the reported reliability and validity metrics, as well as a distinct analysis for adults and children.

### Risk of Bias Assessment

It is important for a systematic review to assess the risk of bias of reports that are included [26]. Risk of bias for the current systematic review will be assessed using the PROBAST (Prediction Model Risk of Bias Assessment Tool) [31]. PROBAST is a risk of bias tool specific to prediction model studies and has 4 domains: participants, predictors, outcome, and analysis [31].

Prior to the risk of bias assessment, the evaluation will be pilot tested by the study’s primary investigators. Research assistant team members will receive training, and their initial data will be checked against assessments completed by the study’s primary investigators. If further explanation and instruction are necessary, remediation will be conducted. Once the study’s primary investigators and research assistant team members are confident with the risk of bias assessment pilot, the phase will proceed. Risk of bias assessment will occur in duplicate by at least 2 independent team members. If disagreements occur, the team will meet to adjudicate the discrepancies, where each team member will have the opportunity to discuss the rationale behind the initially assigned scores and then come to a consensus on the final score.

## Results

We applied for funding to the University of Nevada, Las Vegas Sports Innovation Initiative Catalyst Grant Funding Program in March 2025 and received notice of the award in June 2025. The funding duration is a single year, ending in June 2026. Additionally, we applied for funding to the National Association for Kinesiology in Higher Education in June 2025 and received notice of the Hellison Interdisciplinary Research Grant award in August 2025.

Following the granting of these awards, the team deliberated and agreed upon the protocol presented here. Details are available in PROSPERO, an online international prospective register of systematic reviews. The protocol was created on June 30, 2025, and registered on July 11, 2025 (PROSPERO CRD420251084400).

The databases listed in this review (Figure 1) were searched using the search strategy string between August 1, 2025, and August 8, 2025. Training of team members began on September 3, 2025, and concluded on October 20, 2025.

## Discussion

It is hypothesized that gender-diverse individuals will be underrepresented or entirely absent in the literature used to generate RMR prediction equations. The research team anticipates that there will be great heterogeneity in research methods in the collection of RMR. We expect that such literature lacks clear definitions for sex and gender and may be using these terms interchangeably [7] and/or incorrectly. Furthermore, while not the primary aim of this systematic review, we anticipate underrepresentation or underreporting to be present for other key determinants of health (eg, socioeconomic status, race, etc) [32,33].

If our primary hypothesis is correct, our findings will highlight a key limitation in the RMR prediction equation literature [34]; our work could provide guidance for future researchers working in the area. Moreover, we hope to outline the variety of RMR methodologies used in key prediction equations for researchers considering collecting similar data. Ideally, clear definitions of the terms sex and gender would be used appropriately at the study design phase and throughout the duration of the investigation [35], culminating in the creation of RMR prediction equations that intentionally apply to a more inclusive segment of the population.

Many RMR prediction equations use binary sex as an input to derive estimates [1-4,36-39]; however, it is unknown whether gender-diverse individuals have been included. As noted previously, basing the estimate on sex may be inherently flawed, due to high intrasex variability among male participants compared with female participants [5,6]. This systematic review will critically examine the recruitment and level of inclusion of people from various sexes and genders involved as participants in studies generating RMR prediction equations.

It is expected that the main findings and implications of this systematic review will be disseminated in a variety of formats. These include peer-reviewed publications, presentations, and continuing education workshops. The intention is to complete the systematic review process by December 2026 and submit the narrative synthesis for publication in a peer-reviewed academic journal by July 2027. At the write-up stage, the reporting of this systematic review will be guided by the standards of the PRISMA Statement [40]. This review will identify gaps in the inclusivity of current RMR prediction equations, outline the heterogeneity of RMR research methods, and assess whether terminology, reporting, and interpretations regarding sex and gender are used appropriately [15], all of which should inform future research and clinical applications.

## Abbreviations

PECOS: population, exposure, comparator, outcome, and study design
PRISMA: Preferred Reporting Items for Systematic Reviews and Meta-Analyses
PROBAST: Prediction Model Risk of Bias Assessment Tool
RMR: resting metabolic rate
SAGER: Sex and Gender Equity in Research

## References

[1] Mifflin MD, St Jeor ST, Hill LA, Scott BJ, Daugherty SA, Koh YO (1990). A new predictive equation for resting energy expenditure in healthy individuals. Am J Clin Nutr.

[2] Owen OE, Kavle E, Owen RS, Polansky M, Caprio S, Mozzoli MA, Kendrick ZV, Bushman MC, Boden G (1986). A reappraisal of caloric requirements in healthy women. Am J Clin Nutr.

[3] Owen OE, Holup JL, D'Alessio DA, Craig ES, Polansky M, Smalley KJ, Kavle EC, Bushman MC, Owen LR, Mozzoli MA (1987). A reappraisal of the caloric requirements of men. Am J Clin Nutr.

[4] Harris JA, Benedict FG (1918). A biometric study of human basal metabolism. Proc Natl Acad Sci U S A.

[5] Halsey LG, Careau V, Pontzer H, Ainslie PN, Andersen LF, Anderson LJ, Arab L, Baddou I, Bedu-Addo K, Blaak EE, Blanc S, Bonomi AG, Bouten CV, Bovet P, Buchowski MS, Butte NF, Camps SG, Close GL, Cooper JA, Das SK, Cooper R, Dugas LR, Ekelund U, Entringer S, Forrester T, Fudge BW, Goris AH, Gurven M, Hambly C, Hamdouchi AE, Hoos MB, Hu S, Joonas N, Joosen AM, Katzmarzyk P, Kempen KP, Kimura M, Kraus WE, Kushner RF, Lambert EV, Leonard WR, Lessan N, Martin CK, Medin AC, Meijer EP, Morehen JC, Morton JP, Neuhouser ML, Nicklas TA, Ojiambo RM, Pietiläinen KH, Pitsiladis YP, Plange-Rhule J, Plasqui G, Prentice RL, Rabinovich RA, Racette SB, Raichlen DA, Ravussin E, Reynolds RM, Roberts SB, Schuit AJ, Sjödin AM, Stice E, Urlacher SS, Valenti G, Van Etten LM, Van Mil EA, Wilson G, Wood BM, Yanovski J, Yoshida T, Zhang X, Murphy-Alford AJ, Loechl CU, Luke AH, Rood J, Sagayama H, Schoeller DA, Westerterp KR, Wong WW, Yamada Y, Speakman JR (2022). Variability in energy expenditure is much greater in males than females. J Hum Evol.

[6] Alcantara JM, Osuna-Prieto FJ, Plaza-Florido A (2022). Associations between intra-assessment resting metabolic rate variability and health-related factors. Metabolites.

[7] Webster L (2021). “Ties that bind”: the continued conflation of sex, sexuality and gender. J Lang Sex.

[8] Coen S, Banister E What a difference sex and gender make: a gender, sex and health research casebook. Canadian Institutes of Health Research Institute of Gender and Health.

[9] Richards C, Bouman WP, Seal L, Barker MJ, Nieder TO, T'Sjoen G (2016). Non-binary or genderqueer genders. Int Rev Psychiatry.

[10] O'Neill JE, Corish CA, Horner K (2023). Accuracy of resting metabolic rate prediction equations in athletes: a systematic review with meta-analysis. Sports Med.

[11] Frankenfield D, Roth-Yousey L, Compher C (2005). Comparison of predictive equations for resting metabolic rate in healthy nonobese and obese adults: a systematic review. J Am Diet Assoc.

[12] Sabounchi NS, Rahmandad H, Ammerman A (2013). Best-fitting prediction equations for basal metabolic rate: informing obesity interventions in diverse populations. Int J Obes (Lond).

[13] Frankenfield D, Hise M, Malone A, Russell M, Gradwell E, Compher C (2007). Prediction of resting metabolic rate in critically ill adult patients: results of a systematic review of the evidence. J Am Diet Assoc.

[14] Campos TA, Mariz VG, Mulder AP, Curioni CC, Bezerra FF (2024). Adequacy of basal metabolic rate prediction equations in individuals with severe obesity: a systematic review and meta-analysis. Obes Rev.

[15] Heidari S, Babor TF, De Castro P, Tort S, Curno M (2016). Sex and gender equity in research: rationale for the SAGER guidelines and recommended use. Res Integr Peer Rev.

[16] Siddaway AP, Wood AM, Hedges LV (2019). How to do a systematic review: a best practice guide for conducting and reporting narrative reviews, meta-analyses, and meta-syntheses. Annu Rev Psychol.

[17] Davis DW, Carrier B, Cruz K, Barrios B, Landers MR, Navalta JW (2022). A systematic review of the effects of meditative and mindful walking on mental and cardiovascular health. Int J Exerc Sci.

[18] Carrier B, Barrios B, Jolley BD, Navalta JW (2020). Validity and reliability of physiological data in applied settings measured by wearable technology: a rapid systematic review. Technologies.

[19] Gardner C, Navalta JW, Carrier B, Aguilar C, Perdomo Rodriguez J (2023). Training impulse and its impact on load management in collegiate and professional soccer players. Technologies.

[20] Thomas JD, Flay BR, Cardinal BJ (2018). Are physical activity resources understandable as disseminated? A meta-analysis of readability studies. Quest.

[21] Thomas JD, Wong JC, Hockert RF, Wu YS, Kennedy W (2025). It is not clear how well physical activity promotion materials are understood: findings from a rapid literature review. Top Exerc Sci Kinesiol.

[22] Pitta RM, Montenegro CG, Rica RL, Bocalini DS, Tibana RA, Prestes J, Stone WJ, Figueira Jr. AJ Jr (2019). Comparison of the effects of linear and non-linear resistance training periodization on morphofunctional capacity of subjects with different fitness levels: a systematic review. Int J Exerc Sci.

[23] Melo GL, Neto IV, da Fonseca EF, Stone W, Nascimento DD (2022). Resistance training and Down syndrome: a narrative review on considerations for exercise prescription and safety. Front Physiol.

[24] Stone WJ, Tolusso DV, Duchette C, Malone G, Dolan A (2023). Eccentric resistance training with neurological conditions: a meta analysis. Gait Posture.

[25] Morgan RL, Whaley P, Thayer KA, Schünemann HJ (2018). Identifying the PECO: a framework for formulating good questions to explore the association of environmental and other exposures with health outcomes. Environ Int.

[26] Boland A, Cherry G, Dickson R (2017). Doing a Systematic Review: A Student's Guide.

[27] Freelon D (2013). ReCal OIR: ordinal, interval, and ratio intercoder reliability as a web service. Int J Internet Sci.

[28] Murphy J, Caldwell AR, Warne JP (2025). Reflections on conducting a large replication project in sports and exercise science. Sports Med.

[29] Goodman A, Pollock M (2008). Exposing race as an obsolete biological concept. Everyday Antiracism: Getting Real about Race in School.

[30] Flanagin A, Frey T, Christiansen SL, AMA Manual of Style Committee (2021). Updated guidance on the reporting of race and ethnicity in medical and science journals. JAMA.

[31] Wolff RF, Moons KG, Riley RD, Whiting PF, Westwood M, Collins GS, Reitsma JB, Kleijnen J, Mallett S (2019). PROBAST: a tool to assess the risk of bias and applicability of prediction model studies. Ann Intern Med.

[32] Jo J, Williams KL, Wallace J, Anand M, Anesi T, Brewer C, Burns C, Hefley WF, St Julien Z, Tang AR, Zuckerman SL, Terry DP, Yengo-Kahn AM (2024). Systematic review examining the reporting of race and ethnicity in sport-related concussion studies. J Athl Train.

[33] French MT, Cardinal BJ (2021). Content analysis of equity, diversity, and inclusion in the Recreational Sports Journal, 2005–2019. Recreat Sports J.

[34] Pandit A, Tran TB, Letton M, Cowley E, Gibbs M, Wewege MA, Hagstrom AD (2023). Data informing governing body resistance-training guidelines exhibit sex bias: an audit-based review. Sports Med.

[35] Navalta JW, Davis DW, Thomas JD, Stone WJ (2025). Editorial: a step-by-step statistical decision framework for a gender-inclusive approach in sport and exercise science research. Int J Exerc Sci.

[36] Van Hooren B, Cox M, Rietjens G, Plasqui G (2023). Determination of energy expenditure in professional cyclists using power data: validation against doubly labeled water. Scand J Med Sci Sports.

[37] Freire R, Pereira GR, Alcantara JM, Santos R, Hausen M, Itaborahy A (2022). New predictive resting metabolic rate equations for high-level athletes: a cross-validation study. Med Sci Sports Exerc.

[38] Jagim AR, Camic CL, Askow A, Luedke J, Erickson J, Kerksick CM, Jones MT, Oliver JM (2019). Sex differences in resting metabolic rate among athletes. J Strength Cond Res.

[39] ten Haaf T, Weijs PJ (2014). Resting energy expenditure prediction in recreational athletes of 18-35 years: confirmation of Cunningham equation and an improved weight-based alternative. PLoS One.

[40] Page MJ, McKenzie JE, Bossuyt PM, Boutron I, Hoffmann TC, Mulrow CD, Shamseer L, Tetzlaff JM, Akl EA, Brennan SE, Chou R, Glanville J, Grimshaw JM, Hróbjartsson A, Lalu MM, Li T, Loder EW, Mayo-Wilson E, McDonald S, McGuinness LA, Stewart LA, Thomas J, Tricco AC, Welch VA, Whiting P, Moher D (2021). The PRISMA 2020 statement: an updated guideline for reporting systematic reviews. BMJ.

